# *KCNQ1* G219E and *TRPM4* T160M polymorphisms are involved in the pathogenesis of long QT syndrome: A case report

**DOI:** 10.1097/MD.0000000000024032

**Published:** 2021-01-15

**Authors:** Yang Zhao, Min Feng, Lu-Xiang Shang, Hua-xin Sun, Xian-Hui Zhou, Yan-Mei Lu, Ling Zhang, Qiang Xing, Yao-dong Li, Bao-Peng Tang

**Affiliations:** a Department of Cardiology, First Affiliated Hospital, Xinjiang Medical University, Urumqi, Xinjiang; b Department of Cardiology, Frontier Defence Force General Hospital of Armed Police, Shenzhen, China.

**Keywords:** human induced pluripotent stem cells, long QT syndrome, potassium voltage-gated channel subfamily Q member 1, transient receptor potential cation channel subfamily M member 4

## Abstract

**Rationale:**

Long QT syndrome (LQTS) is an inheritable disease characterized by prolonged QT interval on the electrocardiogram. The pathogenesis of LQTS is related to mutations in LQTS-susceptible genes encoding cardiac ion channel proteins or subunits.

**Patient concerns:**

Here, we reported a 37-year-old female Uygur patient with palpitation and loss of consciousness.

**Diagnoses:**

At the time of admission, a 12-lead electrocardiogram showed a QTc interval of 514 ms. Genetic analysis revealed *KCNQ1* G219E and *TRPM4* T160M mutations.

**Interventions:**

Although beta-blockers remain the mainstay in treating LQTS, the patient underwent implantation of an automatic cardioverter defibrillator due to life-threatening arrhythmias.

**Outcomes:**

To explore the effect of the calcium ion antagonist verapamil on ion channels, we generated human induced pluripotent stem cell cardiomyocytes (hiPSC-CMs) from the peripheral blood mononuclear cells of the patient. The changes of action potential duration in response to verapamil were observed.

**Lessons:**

Our results showed that patient-derived hiPSC-CMs could recapitulate the electrophysiological features of LQTS and display pharmaceutical responses to verapamil.

## 1 Introduction

Long QT syndrome (LQTS) is an inherited cardiac disorder characterized by QT interval prolongation on the electrocardiogram (ECG), leading to occurrence of episodic syncope or sudden cardiac death (SCD).^[1,2]^ Proper treatment in patients with LQTS can dramatically reduce the mortality rate from ∼21% within 1 year since the first syncope to 1% at 15-year follow-up.^[3,4]^ Thus, early diagnosis and intervention are critical in improving the overall survival of patients with LQTS.

The pathogenesis of LQTS has been related to the abnormal function of ion channels resulting from genetic alterations in LQTS-susceptibility genes.^[5–7]^ Genetic testing provides valuable information that may facilitate early clinical intervention in LQTS. Over the past few decades, hundreds of LQTS-causing mutations have been identified in at least 15 LQTS-susceptible genes encoding cardiac ion channels or adaptor proteins.^[8]^ Mutations in three major genes, including *KCNQ1*, *KCNH2*, and *SCN5A*, are responsible for nearly 80% of diagnosed cases,^[9,10]^ whereas the mutations responsible for LQTS cases without these major ones remain elusive. Recently, transient potential melastatin 4 gene (*TRPM4*) encoding an intracellular Ca^2+^-activated non-selective cation channel has been involved in LQTS pathogenesis.^[11]^ TRPM4 channels depolarize the membrane by allowing Na^+^ influx at negative membrane potentials, whereas repolarize the membrane by permitting K^+^ efflux at positive membrane potentials, playing an important role in the shape of action potential (AP).^[12]^ TRPM4 participates in sensing intracellular Ca2+ by changing cell membrane potential, affecting voltage-dependent Ca2+ channels (VGCC) and non-voltage-dependent Ca2+ channels (NVDCC),^[13,14]^ and the intracellular Ca2+ concentration is 0.4 to 9.8 At nmol/L, TRPM4 is activated, and the activation process is regulated by ATP, calcium-calmodulin and PKC.^[15]^

Due to the electrophysiological properties of cardiomyocytes (CMs) that vary from species to species, human induced pluripotent stem cells (hiPSCs) are helpful in testing patient-specific therapies for cardiovascular diseases. In previous studies, investigators have generated hiPSCs from fibroblasts and peripheral blood mononuclear cells (PBMCs) from individual patients and have induced them to differentiate into functional CMs^[16–18]^ that can recapitulate the electrophysiological features of LQTS and respond to putative therapeutic agents.^[16–20]^

## 2 Case presentation

A 37-year-old female Uygur patient was referred to our hospital for palpitation and loss of consciousness. She had a family history of SCD, a history of intermittent palpitation, and 3 syncope events during the past 2 years. The patient was diagnosed with congenital heart disease based on electrocardiography and echocardiography in another hospital. She had been treated with atropine and propranolol for 1 year after the diagnosis of LOTS. The medications were discontinued in the past year, and the above-mentioned symptoms did not appear until a recent relapse. After this relapse, she was brought to a local hospital where ECG revealed a prolonged QT interval (500 ms). Then, she was referred to our hospital. The patient provided written informed consent.

**Figure 1:**
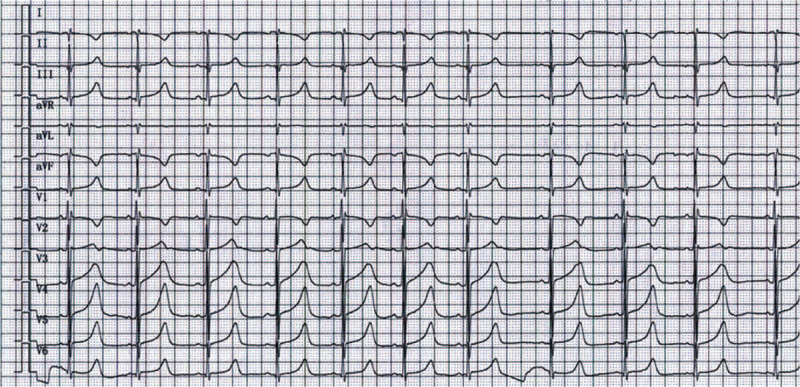
Electrocardiogram (ECG) of the proband with long QT syndrome (LQTS). A 37-year-old Uygur woman with dizziness and palpitations was diagnosed with LQT1 during the clinical evaluation. Surface ECG showed a prolonged QT interval (QT interval corrected for heart rate [QTc], 514 ms).

In the emergency department before any treatment, ECG showed prolonged QT (506 ms) and QTc (514 ms) intervals (Fig. 1). Electrical cardioversion at 150J was performed twice for rhythm control, and continuous infusion of Esmolol was carried out to prevent arrhythmia. Then, the patient recovered from unconsciousness and was admitted to the ward with the diagnosis of LQTS and ventricular tachycardia (VT). One day after admission, she experienced a sudden loss of consciousness. ECG monitoring revealed recurrent episodes of premature ventricular contractions, VT, ventricular fibrillation, and apical torsional VT; 24-h dynamic ECG showed sinus rhythm, frequent ventricular premature beats, paired ventricular premature complexes, short bursts of VT, and ventricular fibrillation. Continuous chest compression and electrical defibrillation at 200J biphasic wave were performed immediately. The sinus rhythm was recovered 6 min later, and the patient regained consciousness. Due to life-threatening arrhythmias, the patient underwent implantation of an automatic cardioverter defibrillator.

## 3 Genetic testing of the proband and her family members

**Figure 2:**
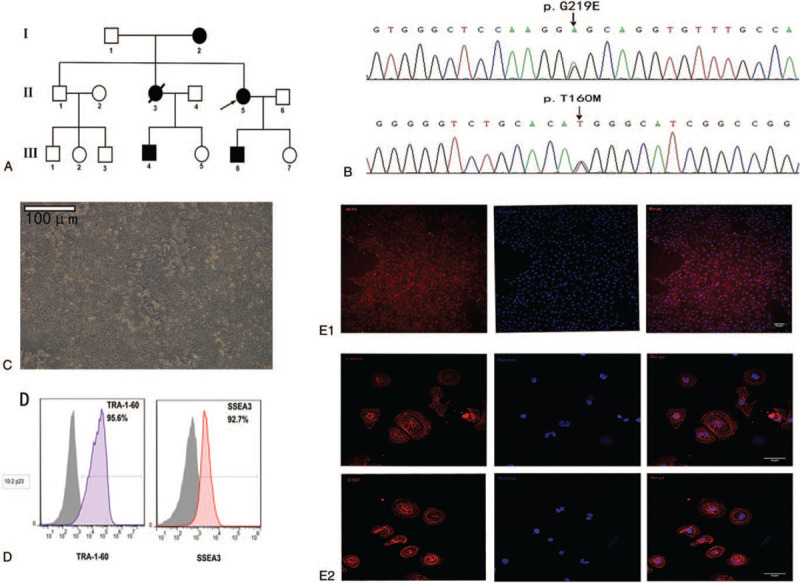
Generation and characterization of human induced pluripotent stem cells (hiPSCs). (A) Pedigree of the proband with LQTS. Screening of the proband's family revealed prolonged QT in her mother (Patient I-2), her son (Patient III-1), and her elder sister's son (Patient III-3). Squares and circles indicate male and female family members, respectively; solid, open, and line-crossed symbols indicate LQTS diagnosed, unaffected, and deceased family members, respectively. (B) Whole exome sequencing was performed on DNA isolated from peripheral blood samples of the proband and her family. (C) Embryonic stem cell-like clusters. (D) Flow cytometry analysis was performed to determine the percentage of iPSCs expressing the pluripotency surface markers SSEA3 and TRA-1-60. (E1 and E2) Immunofluorescent staining of the pluripotency marker OCT4 in iPSCs. Scale bar.

This study was approved by the Ethics Committee of Xinjiang Medical University First Affiliated Hospital (Xinjiang, China). All participants provided written informed consent before blood collection. Whole exome sequencing of DNA from PBMCs of the patient showed that the *TRPM4* gene had a T160M mutation resulting from a single base exchange in the fifth exon (479C→T). A G219E missense mutation was also found in the *KCNQ1* gene resulting from a heterozygous single base exchange in the fourth exon (656G→A) (Fig. 2B). Subsequent screening of the members of the proband's family revealed prolonged QT in her mother (QTc 516 ms), son (QTc 506 ms), and elder sister's son (QTc 520 ms). The proband and her mother had recurrent syncope. The deceased elder sister experienced sudden death (Fig. 2A).

## 4 Generation and characterization of patient-specific iPSCs

Blood samples were obtained from the proband with LQT1 and her healthy brother. After generation of iPSCs, immunofluorescence staining, and flow cytometry were performed to examine the presence of pluripotency-associated proteins. The iPSC clones were further characterized by flow cytometry to determine the percentage of cells expressing the pluripotency surface markers SSEA and TRA-1–60 (Fig. 2D). The blood sample of the proband was reprogrammed with non-integrating Sendai virus vectors carrying Yamanaka factors (OCT4, SOX2, KLF4, and c-MYC). At day 15 post-transduction, embryonic stem cell (ESC)-like clusters started to appear (Fig. 2C). Immunofluorescent staining showed that the iPSC clones expressed the intracellular pluripotency marker OCT4 (Fig. 2E1). The positive staining of cardiomyocyte markers cTNT and α-actinin suggested that the iPSCs were differentiated into CMs (Fig. 2E2).

**Figure 3:**
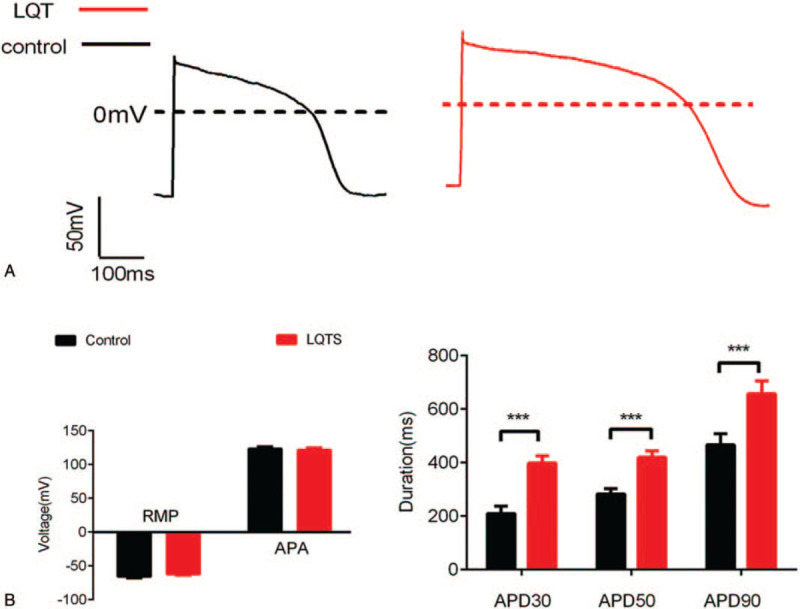
Electrophysiological characterization of hiPSC-derived cardiomyocytes (hiPSC-CMs). Spontaneous and paced (1 Hz) action potentials (AP) (A), resting membrane potential/AP amplitude, and APD90/APD50/APD30 (B) were determined in patient and control hiPSC derived-CMs, respectively. APA = action potential amplitude, APD = action potential duration, RMP = resting membrane potential.

**Figure 4:**
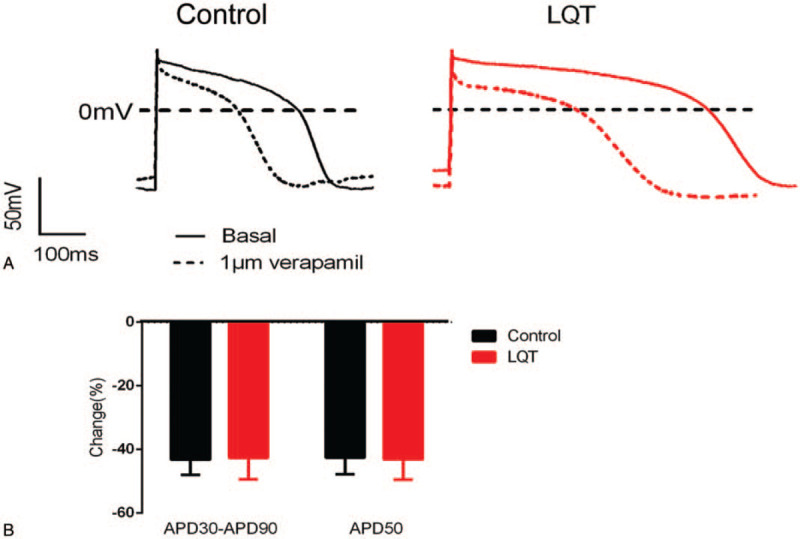
Drug response. HiPSC-MCs derived from the patient and control were treated with verapamil (1 μM). AP (A) and APD30/APD50/APD90 (B) in patient- and control-derived hiPSC-MCs at a 1-Hz stimulation rate were determined. Data are mean ± standard error of the mean (SEM).

To evaluate whether the CMs derived from patient-specific iPSCs recapitulate the disease phenotype, we recorded the APs in single cells. Both spontaneously beating cells dissociated from LQTS and control explants responded to pacing and generated APs. Spontaneous and paced (1 Hz) APs were recorded in patient and control hiPSC derived-CMs. Pacing was conducted to standardize cell contraction rate and minimize the effects of varying cell contraction rates on AP duration (APD). Patient and control hiPSC-CMs were identified by their characteristic AP properties (Fig. 3A). We observed significantly prolonged APD90, APD50, and APD30 in patient hiPSC-CMs compared with control cells during spontaneous contraction (Fig. 3B), suggesting that patient hiPSC-CMs can recapitulate the electrophysiological features of LQTS as measured by patch clamp. In addition, verapamil exposure significantly decreased the QT interval in both control and patient hiPSC-CMs, indicating that hiPSC-CMs were responsive to therapeutic agents (Fig. 4A). The differences in APD between patient and control cells for A-like and N-like hiPSC-CMs were not statistically significant (Fig. 4B).

## 5 Discussion

Mutations in *KCNQ1*, *KCNH2*, and *SCN5A* are responsible for ∼80% of cases diagnosed with LQTS, leaving room for the discovery of additional causal genes for this disease.^[9,10]^ In this study, we identified missense mutations G219E and T160M in *KCNQ1* and *TRPM4*, respectively, in a proband from a family affected by LQTS and reprogrammed PBMCs from the proband and one healthy family member to generate functional hiPSCs-CMs. These myocytes showed expression of specific markers and electrophysiological features. In addition, we observed disease-specific prolonged APD resulting from the trafficking defects of KCNQ1 and pharmacological response to verapamil (a calcium channel blocker) treatment in hiPSCs-CMs. Our data suggest that patient-specific hiPSC-CMs can be derived from somatic cells, recapitulate the electrophysiological features of the disorder, and display pharmaceutical responses to therapeutic drugs, serving as a predictive system for drug assessment in LQTS treatment.

The electrophysiological properties of CMs vary from species to species, and animal models can hardly simulate the electrophysiological properties of human cells. For example, downregulation of complementary rapid and slow delayed rectifier K^+^ currents has been observed in a transgenic animal model, but not in patient-specific myocytes with R190Q-KCNQ1 mutations. This discrepancy may be mutation-dependent or related to the model, highlighting the importance of using a model that can mimic the human condition. Patient-derived hiPSCs provide a promising approach for understanding the pathogenesis and testing patient-specific therapies for cardiovascular diseases in vitro. The pluripotency of hiPSCs and the unlimited number of induced CMs can facilitate a high-throughput drug screening.

*KCNQ1* gene mutation reduces the main component of myocardial repolarization outward current Iks, delays ventricular repolarization, and thereby prolongs QT interval. Adrenergic stimulation in healthy individuals during vigorous exercise can increase repolarization outward current while reducing APD via increased Iks, Ica-1, Icl, and Icl-camp, leading to heart rate elevation and QTc shortening. In contrast, the Iks ion channel with structural abnormalities due to *KCNQ1* mutation loses adrenaline responsiveness, leading to APD prolongation via increased inward current and decreased outward current. A previous study using a LQT1 model with LQT2 or rapid pacing showed that verapamil can significantly shorten QT interval and APD.^[21]^ In this study, the APD of both normal control and LQT1 patient hiPSCs-derived CMs were significantly decreased in response to 1 μM verapamil, suggesting that these cells may display pharmaceutical responses to therapeutic drugs and could be used in drug screening for LQT therapy.

In conclusion, we reported a LQTS case with *KCNQ1* G219E and *TRPM4* T160M polymorphisms, and generated patient-specific hiPSCs from PBMCs. These cells were further directed to differentiate into functional hiPSCs-CMs. The AP changes in response to ion channel blockers were measured to evaluate pharmacological responses. Our data suggest that patient-specific hiPSC-CMs can be derived from somatic cells and may serve as a predictive system for drug assessment in LQTS treatment.

## Abbreviations

Abbreviations: AP = action potential, APD = AP duration, CMs = cardiomyocytes, ECG = electrocardiogram, ESC = embryonic stem cell, hiPSCs = human induced pluripotent stem cells, LQTS = long QT syndrome, NVDCC = non-voltage-dependent Ca2 + channels, PBMCs = peripheral blood mononuclear cells, SCD = sudden cardiac death, TRPM4 = transient potential melastatin 4 gene, VT = ventricular tachycardia.
